# Antiretroviral therapy non-adherence among children living with HIV in Dire Dawa, Eastern Ethiopia: a case-control study

**DOI:** 10.1186/s12887-022-03697-1

**Published:** 2022-11-10

**Authors:** Melkamu Merid Mengesha, Mulugeta Embibel, Tesfaye Gobena, Abayneh Tunje, Degu Jerene, Inger Kristensson Hallström

**Affiliations:** 1 Unit of Epidemiology and Biostatistics, School of Public Health, College of Medicine and Health Sciences, Arba Minch University, Arba Minch, Ethiopia; 2 Urban Health facilities of Dire Dawa Regional Health Bureau, Dire Dawa, Ethiopia; 3 Department of Environmental Health Sciences, College of Health and Medical Sciences, Haramaya University, Harar, Ethiopia; 4 KNCV Tuberculosis Foundation, Den Haag, The Netherlands; 5 Child and Family Health, Department of Health Sciences, Faculty of Medicine, Lund University, Lund, Sweden

**Keywords:** Non-adherence to ART, HIV status disclosure, Children, and viral load

## Abstract

**Background:**

In 2018, nearly 90% of the global children living with human immunodeficiency virus (HIV) were in sub-Saharan Africa (SSA). Compared to the adult population, antiretroviral therapy (ART) coverage among children was limited. However, adherence remained a problem among children though they had limited access to ART. This study was conducted to identify the risk factors of non-adherence to ART among children aged 6 to 17 years.

**Methods:**

This case-control study was conducted in 2020 using data obtained from clinical record reviews and self-reported data from 272 caregivers of HIV-infected children aged 6–17 years. Cases and controls represented children with poor versus children with good adherence to ART, respectively. Good adherence was defined based on a past 30-day physician adherence evaluation of taking ≥ 95% of the prescribed doses. Binary logistic regression was used to identify factors associated with non-adherence to ART. All statistical tests are defined as statistically significant at P-values < 0.05.

**Results:**

Of the 272 children, for whom data were obtained, 78 were cases and 194 were controls; females accounted for 56.3%, 32% attended secondary school, and for 83.1%, the reporting caregivers were biological parents. Non-adherent children had higher odds of association with the following risk factors: a caregiver who is a current substance user (aOR = 2.87, 95% CI: 1.44, 5.71), using AZT-and ABC-based regimen compared to the TDF-regimen (AZT-based, aOR = 4.12, 95% CI: 1.43, 11.86; ABC-based, aOR = 5.58, 95% CI: 1.70, 18.30), and had an increase in viral load from baseline compared to those remained undetectable (remained at or decreased to < 1000, aOR = 4.87, 95% CI: 1.65, 14.33; remained at ≥ 1000, aOR = 9.30, 95% CI: 3.69, 23.46). In contrast, non-adherent children had 66% lower odds of being at early adolescent age compared to 6–9 years old (10–14 years, aOR = 0.34, 95% CI: 0.12, 0.99) and had 70% lower odds of being aware of their HIV status (aOR = 0.30, 95% CI: 0.13, 0.73).

**Conclusion:**

Technical support to caregivers to build disclosure self-efficacy, identifying the appropriate regimen for children, counseling on viral load suppression on subsequent visits, and helping caregivers avoid or reduce substance use may help improve the problem of children’s non-adherence to ART.

## Background

Human immunodeficiency virus (HIV) remains a serious global public health concern with low and middle-income countries (LMICs) sharing the most burden [1]. In 2018, 9 out of 10 children (< 18 years) (2.52 million out of 2.8 million global total) living with HIV are in sub-Saharan Africa(SSA) [1]. Concerning to access to antiretroviral therapy (ART), compared to the adult population living with HIV, ART coverage and access to treatment were limited among children [2]. For example, the global UNAIDS (The Joint United Nations Programme on HIV and AIDS) statistics reported that only 54% of children had access to ART [2]. Despite a sustained increase in ART coverage in SSA countries, the percent of children receiving ART in Ethiopia is still below 40% [3].

Globally in 2021, 28.7 people living with HIV were receiving ART, and the global coverage in 2021 was 75% [66–85%]. Only 52% [42–65%] of children (0–14 years old) were receiving ART at the end of 2021 [4]. On top of the limited access to ART treatment among children, adherence is a problem particularly among adolescents (10–19 years) living with HIV (ALHIV) [5]. At a global level, only 62.3% of ALHIV were adherent to therapy [6]. In SSA, a narrative review of adherence to ART among adolescents by Adejumo et al. reported that only < 86% took 95% of their prescribed pills during a specified duration [5]. Although adherence levels ≥ 80% have been associated with treatment success [7], optimal adherence (≥ 95%) is widely considered desirable for viral suppression and prevention of ART resistance [8].

Non-adherence to ART among children remains the single most significant challenge in HIV/AIDS care and treatment. They face unique challenges related to adherence as they are still largely dependent on their caregivers to take their medications, and even at some times, may refuse to take the medications, especially young children, as the reason for such medication may not have been disclosed to them [9, 10]. Without adequate adherence to ART, children are at a greater risk of viral resistance to the available drugs, treatment failure, immunologic decline resulting in opportunistic infections, and HIV disease progression [11, 12].

Several adherence studies, particularly in SSA, are often focused on adults despite children having unique challenges concerning adherence to ART [5, 13]. A systematic review by Ammon et al. reported a complex web of factors affecting children including stigma, ART side-effects, lack of assistance, and forgetfulness as barriers to adherence [14]. Similarly, the same study reported on adherence facilitators including caregiver support, peer group support, and knowledge of ART status [14]. Only a few published studies reported factors associated with adherence to ART among children in Ethiopia [15–18] limiting the availability of the current evidence base for interventions that apply to the Ethiopian context in the era of the 90-90-90 target to reduce HIV by 2020 [19]. These studies conducted in Ethiopia measured adherence based on caregiver self-report, adherence reports of younger children (≤ 14 years), and are cross-sectional in design [16–18]. Findings on factors associated with adherence include those related to the caregiver (young adults, marital status, education, knowledge on ART, not on treatment themselves, and substance use), child (knows his/her HIV status), health facility (counseling before ART initiation and proximity to health facility) and medication [15–18].

This current study used a physician-documented adherence assessment, employed a better study design and data collected covered different age ranges of children, 6 to 17 years. In addition to the factors that previously have been reported on the association with adherence to ART, our study assessed the orphan status, ART regimen, and whether a change in the viral load count during follow-up visits encouraged adherence to ART. The viral load count services (initial measurement at six months after ART initiation and then repeated every 12 months) were scaled up and become routine in HIV care in Ethiopia only recently in 2016 [20].

## Methods and materials

### Study area and periods

The study was conducted in ten urban healthcare facilities (two private and eight public health facilities) in Dire Dawa City Administration, eastern Ethiopia, and located 515 km from Addis Ababa. When this study was conducted, the total population of Dire Dawa was 506,936 (51.0% were females), according to the Dire Dawa Regional Health Bureau Plan and Program Office. 68% of the population was urban dwellers and the rest 32% of the population were living in rural areas. There were 16 ART service-providing sites in Dire Dawa (10 public and 6 private health facilities) a total of 6515 PLHIV were currently receiving ART (6,148 were adults and 367 were children). This current study was conducted in ten selected healthcare facilities (two hospitals each from private and public health facilities and six health centers). Data were collected during the periods from June 10, 2020, to August 15, 2020.

### Study design

A case-control study was conducted to identify risk factors of non-adherence to ART among children who received care and treatment in public and private health facilities in Dire Dawa.

#### Population

All caregivers of HIV-infected children who were aged 6–17 years, currently active on ART when the study was conducted, and have used ART for at least six months participated in the study. Cases were defined when a child/adolescent was non-adherent to ART based on clinical document review. Similarly, controls were defined when a child/or an adolescent was adherent to ART.

#### Sample size determination

The sample size was determined using the OpenEpi software V3.01 with the following assumptions: proportions of children who knew their HIV status (49.4%) [21], 5% level of significance, 80% power, a 1:2 case vs. control ratio, and odds ratio of 2 [22]. Accordingly, the minimum sample size with Fleiss correction was 308 (103 cases and 205 controls). Conducting document reviews of all pediatric ART users in the selected health facilities, we identified 272 eligible subjects after excluding 32 patient cards of children on second-line drugs. Consequently, the final sample size was 272 (78 cases and 194 controls).

#### Sampling technique

Selection of the healthcare facilities was based on the number of active pediatric HIV/AIDS patients on chronic care follow-up. Of the ten selected ART-providing healthcare facilities in Dire Dawa City, four were hospitals (two public and two private hospitals) and six were health centers (primary care units). The number of eligible children per facility was 198 in the four hospitals (139 in Dilchora Referral Hospital; 39 in Sabian Primary Hospital; 10 each in Bilal and DELT private hospitals) and 74 in the remaining six health centers (11 each in Goro and Dechatu Health Centers; 12 in Gendekore Health Center; 13 each in Dire Dawa and Adis Ketema Health Centers; and 14 in Leghare Health Center). We did not do further sampling from the facilities considered; we rather considered all eligible children in this study and reviewed data from their records and caregivers provided self-reported data.

The next appointment dates of all children were identified from computer databases in the pediatric ART clinics of the selected health facilities and ART refilling was conducted monthly. In collaboration with the ART clinic staff, the research team passed a message to caregivers to accompany their child during the next visit or request to visit the health facility if the next appointment date was passed without reaching them. When a child was deemed eligible for inclusion, all caregivers were invited to participate in the study.

### Data collection and variables measurement

Data were collected from caregivers through a face-to-face interview using structured questionnaires and checklists were used to abstract children’s clinical data from medical charts. Data were collected by eight BSc nurses and two data clerks who were oriented about the study objectives and data collection tools, variable measurement, data source, covid-19 prevention protocols, and collection techniques. Data collectors had previously received training on HIV/AIDS care and treatment as part of their on-job professional career development.

The data collected in this study included the caregivers’ socio-demographic and behavioral related data and children’s socio-demographic and clinical data. The socio-demographic and behavioral variables collected **that were** related to caregivers include age, sex, marital status, level of education, and current substance use was measured using a single question, “do you currently use substances like alcohol or khat?” Children’s demographic information collected includes age, sex, education, caregiver’s relationship with the child, disclosure of HIV status, and orphanhood. The disclosure of children’s HIV status was based on the caregiver’s self-report to a ‘yes/no’ response item (“did your child know that s/he was HIV positive?”). The caregiver-child relation was ascertained to identify whether the responding caregiver was a biological parent or other.

Clinical data of eligible HIV-infected children were obtained from the databases in the selected ART clinics. Duration since ART was calculated by subtracting the date of ART initiation from the date of the last refill. Other clinical data collected were daily ART dose, CD4 count (cells/mm^3^), viral load (copies/mL), and WHO clinical stage. As there were significant missing values for CD4 count, we considered the baseline CD4 count (cells/mm^3^) results available. We also took the initial WHO clinical stage documented. HIV viral load testing in routine care, however, has started only recently in 2016 in Ethiopia[20]. In routine HIV care, the first HIV viral load measurement was supposed to be taken at six-month after initiation of ART and repeated at 12 months, and then every 12 months. We took two-time points (the initial and the last measured values) and computed the difference in the HIV viral load between the two-time points.

A case and control status in terms of adherence to ART was determined by observing last month’s physician’s assessment of adherence as reviewed in the medical chart in the ART clinics. Accordingly, cases were children who had a “poor” level of adherence in the past 30 days according to the physician’s evaluation (< 95% of prescribed drugs are consumed) and those who had a “good” level of adherence (≥ 95% of prescribed drugs are consumed) were identified as controls.

One of the co-investigators who supervised the data collection (ME) pre-identified both cases and controls from the respective health facilities by reviewing medical charts in the ART clinics using their unique ART numbers. These unique ART numbers were then communicated to the respective data collectors in the selected health facilities. Finally, self-reported data were collected from caregivers of children with poor (cases) and “good” (controls) levels of adherence. To conceal the case and control status from data collectors, the unique identifiers communicated to them were not labeled with a case-control status.

### Data processing, analysis, and management

The data were entered into Epi-data version 3.1 and exported to Stata version 14 software for analysis. Frequency, percentages, and numerical summary measures were used to present the descriptive findings. Bivariable and multivariable binary logistic regression analyses were conducted to identify factors associated with non-adherence to ART. Variables that had a p-value (p < 0.25) in the bivariable binary logistic regression model are entered in the multivariable model. Model fitness of the final model was checked using the Hosmer-Lemeshow test in Stata (using the post-estimation command: *estat gof*) and it demonstrated a good fit with Pearson chi2 (164) = 182.9 and P-value = 0.149. Adjusted odds ratio (aOR) along with 95% CI was estimated to identify factors associated with non-adherence to ART. Multicollinearity was checked using the variance inflation factor (VIF) (higher VIF suggests the possible existence of collinearity), and in our analysis, the mean VIF was 3.93. Statistical estimates were considered significant at P-value < 0.05.

### Ethical considerations

All methods in the study were performed following the relevant guidelines and regulations, e.g., the Declaration of Helsinki. The ethical approval is obtained from the Haramaya University, College of Health and Medical Sciences, Institutional Health Research Ethics Review Committee (IHRERC) with a reference number of IHRERC/125/2020. The College of Health and Medical Sciences wrote a formal letter of cooperation to the Dire Dawa City Administration Council Health Bureau. Data collection was started after obtaining informed, written, voluntary, and signed consent from the caregivers. Data were collected confidentially without extracting patient-identifying information.

## Results

### Socio-demographic characteristics

A total of 272 caregivers of HIV-infected children participated in this study. The median age of caregivers was 40 years with only 16.5% being below 34 years of age. Female caregivers constituted 72.1% and, of the total caregivers, 41.5% did not have formal schooling. Most of the caregivers, 87.9%, were HIV-positives. There was no significant difference among cases and controls in terms of caregiver’s demographic and HIV status except for the caregiver’s current alcohol use (Table 1).

The median age of the children was 14 years. Of the total children for whom data were obtained, females accounted 56.3%, and 32% attended secondary school. For 83.1%, the reporting caregivers were their biological parents. With regard to knowledge of their HIV status, based on caregivers report, 61.8% knew their HIV status. There was a significant difference among cases and controls in terms of child age, educational status, and HIV status disclosure where cases tend to be younger, not going to school, and not disclosed, respectively (Table 1).

**Table 1:** Socio-demographic characteristics of caregivers and their children in urban health facilities in Dire Dawa, 2020 (n = 272)

Variables			Total		Cases (n = 78)				Controls (n = 194)		Chi2 (df)		P-value			
					N (%)				N (%)							
Caregivers’ characteristics																
Age in years	23–34		45 (16.5)		17(21.8)		28(14.4)				3.36 (2)		0.186			
	35–44		124 (45.6)		37(47.4)		87(44.9)									
	45+		103 (37.9)		24(30.8)		79(40.7)									
Sex	Male		76 (27.9)		20 (25.6)		56 (28.9)				0.29 (1)		0.592			
	Female		196 (72.1)		58 (74.4)		138(71.1)									
Marital status	Married		127 (46.7)		37 (47.4)		90 (46.4)				0.52 (3)		0.915			
	Divorce		43 (15.8)		11 (14.1)		32 (16.5)									
	Widowed		75 (27.6)		21 (26.9)		54 (27.8)									
	Others		27 (9.9)		9 (11.5)		18 (9.3)									
Education	No formal education		113 (41.5)		39 (50.0)		74 (38.1)				4.41 (2)		0.110			
	Grade 1–8		65 (23.9)		19 (24.4)		46 (23.7)									
	Grade 9–12+		94 (34.6)		20 (25.6)		74 (38.1)									
Relationship with child	Biological parent		226 (83.1)		65(83.3)		161(83.0)				0.01 (1)		0.945			
	Non-biological parent		46 (16.9)		13(16.7)		33(17.0)									
HIV status	Positive		239 (87.9)		71 (91.0)		168(86.6)				1.02 (1)			0.312		
	Negative		33 (12.1)		7 (9.0)		26(13.4)									
Current substance use	Yes		110 (40.4)		46 (59.0)		64 (33.0)				15.60 (1)			< 0.001		
	No		162 (59.6)		32 (41.0)		130 (67.0)									
Variables				Total		Cases (n = 78)				Controls (n = 194)		Chi2 (df)			P-value	
Children’s characteristics																
Age in years		6–9		39 (14.3)		22(28.2)		17(8.8)				17.94 (2)			< 0.001	
		10–14		116 (42.7)		31(39.7)		85(43.8)								
		15–17		117 (42.0)		25(32.1)		92(47.4)								
Sex		Male		119 (43.8)		29(37.2)		90(46.4)				1.92 (1)			0.166	
		Female		153 (56.3)		49(62.8)		104(53.6)								
Education		Not going to school		26 (9.6)		16(20.5)		10 (5.2)				16.52 (2)			< 0.001	
		Primary(1–8)		159 (58.5)		44 (56.4)		115 (59.3)								
		Secondary (9–12)		87 (32.0)		18(23.1)		69 (35.6)								
HIV disclosure status		Yes		168 (61.8)		28(35.9)		140(72.2)				30.99 (1)			< 0.001	
		No		104 (38.2)		50(64.1)		54(27.8)								
Orphan status		Not orphaned		238 (87.5)		66 (84.6)		172 (88.7)				0.832 (1)			0.362	
		Orphaned		34 (12.5)		12 (15.4)		22 (11.3)								
Average family income (ETB)		500–2499		156 (57.4)		43 (55.1)		113 (58.3)				0.957 (2)			0.620	
		2500–4999		94 (34.6)		30 (38.5)		64 (33.0)								
		≥ 5000		22 (8.1)		5 (6.1)		17 (8.8)								

chi2 (df) = chi-square degree of freedom; HIV = Human immunodeficiency virus. ETB = Ethiopian Birr

### Clinical characteristics of children on ART

At baseline, 80% of children were on WHO clinical stage-I. With regard to duration on ART, 93.4% were on ART for at least 5-years with 64% taking a single daily ART dose. Comparing initial viral load values at admission against the recent values, for 45.2%, the viral load values remained at greater than 1000 counts or viral load has increased from the level it was not detectable at baseline. At baseline, however, 65.1% had a non-detectable viral load (Table 2).

**Table 2:** Clinical characteristics of children aged 6–17 years in urban health facilities in Dire Dawa, 2020 (n = 272)

Variables		Total , N = 272	Cases (n = 78)		Controls (n = 194)	Chi2(df)	p-value
			N (%)		N (%)		
Duration on ART	< 5 years	18 (6.6)	13 (16.7)		5(2.6)	20.11 (2)	< 0.001
	5–10 years	93 (34.2)	29 (37.2)		64 (32.9)		
	> 10 years	161 (59.2)	36 (46.2)		125 (64.4)		
Daily ART dose	One	174 (64.0)	37 (47.4)		137 (70.6)	12.97 (1)	< 0.001
	Two	98 (36.0)	41(52.6)		57 (29.4)		
Base line CD4 Count (cells/mm^3^)	< 200	14 (5.1)	8 (10.3)		6 (3.1)	8.38 (3)	0.039
	200–499	65 (23.9)	18 (23.1)		47 (24.2)		
	>=500	180 (66.2)	46 (59.0)		134 (69.1)		
	Not recorded	13 (4.8)	6 (7.7)		7(3.6)		
ART regimen backbone	AZT + 3TC	68 (25.0)	34 (43.6)		34 (17.5)	33.78 (2)	< 0.001
	TDF + 3TC	161 (59.2)	25 (32.1)		136 (70.1)		
	ABC + 3TC	43 (15.8)	19 (24.4)		24 (12.4)		
Variables		Total , N = 272	Cases (n = 78)	Controls (n = 194)		Chi2(df)	p-value
			N (%)	N (%)			
Viral load change from baseline values (copies/mL)	Remained ND	91 (33.5)	9 (11.5)	82 (42.3)		28.23 (2)	< 0.001
	No change or become < 1000	58 (21.3)	16 (20.5)	42 (21.7)			
	≥ 1000 or increased from ND	123 (45.2)	53 (68.0)	70 (36.1)			
WHOs clinical stage on admission	Stage-1	217 (79.8)	64(82.1)	153(78.9)		3.32 (2)	0.190
	Stage-2	38 (14.0)	7(8.9)	31(15.9)			
	Stage-3	17 (6.2)	7(8.9)	10(5.2)			
Experienced side effect	Yes	30	9 (11.5)	21 (10.8)		0.029 (1)	0.865
	No	242	69 (88.5)	173 (89.2)			

ART = Antiretroviral therapy; chi2 (df) = chi-square degree of freedom; ND = Not detectable; WHO = World Health Organization; AZT = Zidovudine; 3TC = Lamivudine; TDF = Tenofovir; ABC = Abacavir

### Factors associated with non-adherence to ART

To move variables in the bivariable binary logistic regression model, we are guided by a P-value < 0.25, a model improvement compared to the null model as measured by the − 2log-likelihood value (the lower is the better), and some important variables are considered irrespective of their statistical non-significance in the bivariable model (family income, caregiver’s HIV status, and caregiver’s relationship with the child). Variables with a significant association with non-adherence to ART in the bivariable model are shown in Table 3. The following variables were found independently and significantly associated with non-adherence to ART while controlling for other factors in the multivariable model (except change in viral load from baseline value): caregiver’s current substance use, HIV status disclosure, and ART regimen. When a separate model was run (as part of sensitivity analysis) with and without viral load change from baseline, there was a model improvement (based on the − 2log-likelihood): the log-likelihood value with ‘viral load change in the model’ was − 118.461 versus − 132.953 without. The variables that remained significant in the separate models are the caregiver’s current substance use, child HIV status disclosure, and ART regimen. When viral load change from baseline was added to the previous model, child age and viral load change become significant (Table 3).

Children who were non-adherent to ART had 2.87 times higher odds of having a caregiver who was currently an active substance user compared to a current non-user caregiver (substance use, aOR = 2.87, 95% CI: 1.44, 5.71). Similarly, compared to children taking the ART regimen with TDF + 3TC backbone, non-adherent children had 4.12 and 5.58 higher odds of taking other ART regimens (ART regimen: AZT + 3TC backbone, aOR = 4.12, 95% CI: 1.43, 11.86; ABC + 3TC backbone, aOR = 5.58, 95% CI: 1.70, 18.30). Another important variable related to children’s non-adherence to ART was whether there was a change in viral load from that observed at baseline. Accordingly, compared to children with non-detectable viral load values at two-time points, non-adherent children had 5 to 9 times higher odds of having no change from the initial measured value or increased viral load values (remained at < 1000 copies/mL or decreased to < 1000 copies/mL, aOR = 4.87, 95% CI: 1.65, 14.33; remained at ≥ 1000 copies/mL or moved up from a non-detectable level, aOR = 9.30, 95% CI: 3.69, 23.46). In contrast, child being at early adolescent age and child’s HIV status disclosure were protective against non-adherence to ART (child age, 10–14 years, AOR = 0.28, 95% CI: 0.09, 0.87; HIV status disclosure, AOR = 0.29, 955 CI: 0.12, 0.68) (Table 3).

**Table 3:** Crude and adjusted odd ratios of factors associated with non-adherence to ART among HIV infected children aged 6 -17years old in urban health facilities of Dire D Dawa, 2020 (n = 272)

Variables			cOR, 95% CI		P-value		aOR, 95% CI Model 1^†^		P-value		aOR, 95% CI Model 2^¥^			P-value
Caregiver’s substance use	No		Ref.				Ref.				Ref.			
	Yes		2.92 (1.70, 5.02)		< 0.001		2. 47 (1.31, 4.66)		0.005		2.87 (1.44, 5.71)			0.003
Child age	6–9		Ref.				Ref.				Ref.			
	10–14 years		0.28 (0.13, 0.60)		0.001		0. 62 (0.25, 1.51)		0.290		0.34 (0.12, 0.99)			0.048
	15–17 years		0.21 (0.10, 0.45)		< 0.001		1.44 (0.42, 4.91)		0.557		0.83 (0.21, 3.33)			0.789
HIV status disclosure	No		Ref.				Ref.				Ref.			
	Yes		0.22 (0.12, 0.38)		< 0.001		0.31 (0.14, 0.69)		0.004		0.30 (0.13, 0.72)			0.007
ART dose	Once daily		Ref.		< 0.001		Ref.				Ref.			
	12 hourly (two doses)		2.66 (1.55, 4.58)				0.50 (0.19, 1.34)		0.168		0.41 (0.15, 1.14)			0.086
Monthly family income (in ETB)	<2500		Ref.				Ref.				Ref.			
	2500–4999		1.23 (0.71, 2.15)		0.464		1.43 (0.75, 2.72)		0.279		1.65 (0.82, 3.35)			0.164
	≥ 5000		0.77 (0.27, 2.22)		0.633		0.73 (0.22, 2.38)		0.598		0.76 (0.22, 2.66)			0.669
Relation with child	Biological parent		Ref.				Ref.				Ref.			
	Non-biological parent		0.98 (0.48, 1.97)		0.945		1.52 (0.56, 4.11)		0.410		1.76 (0.59, 5.25)			0.314
Caregiver’s HIV status	Positive		Ref.				Ref.				Ref.			
	Negative		0.64 (0.26, 1.54)		0.315		0.55 (0.16, 1.85)		0.334		0.65 (0.17, 2.52)			0.531
ART regimen backbone	TDF + 3TC		Ref.				Ref.				Ref.			
	AZT + 3TC		5.44 (2.87, 10.30)		< 0.001		5.40 (1.95, 14.98)		0.001		4.12 (1.43, 11.86)			0.009
	ABC + 3TC		4.31 (2.06, 9.01)		< 0.001		5.56 (1.85, 16.73)		0.002		5.58 (1.70, 18.30)			0.005
Viral load change against the first measurement		Remained ND		Ref.				***		***		Ref.		
		Decreased to or remained < 1000		3.47 (1.41, 8.51)		0.007		***		***		4.87 (1.65, 14.33)	0.004	
		Remained at > = 1000 or increased from ND		6.90 (3.18, 14.98)		< 0.001		***		***		9.30 (3.69, 23.46)	< 0.001	

aOR = adjusted odds ratio, cOR = Crude odds ratio, ART = Antiretroviral Therapy, HIV = Human Immunodeficiency, and Virus ND = Not Detectable. ^†^as part of sensitivity analysis, model I was run without a variable called ‘viral load change’ in the model and ^¥^model II was run including it in the final model

## Discussion

This study identified risk factors of non-adherence to ART among children (6–17 years) living with HIV in Dire Dawa, East Ethiopia. While disclosure of HIV status and child being at an early adolescent age reduced the odds of non-adherence to ART, caregiver’s substance use, ART regimen type, and persistent viral load were associated with higher odds of non-adherence to ART.

The finding in this study that disclosure of HIV status was associated with a reduction in non-adherence confirmed previous similar reports from different settings. A study in Ghana on children’s (6–15 years old) adherence to ART reported that disclosure of HIV status was not only associated with improved adherence but also with psychological well-being [23]. With the disclosure of HIV status, children get cleared with many of their previously unanswered questions concerning the reasons why they were taking medications, engage in peer support groups and ask questions for social support, and improve relationships with caregivers [24, 25]. Evidence from a systematic review by Ammon et al. also reported that children who received disclosure of their HIV status earlier, before age 12, had good adherence to ART [14].

The association of age and adherence to ART was not consistent in the literature where some found increasing adherence problems among older children [5, 14, 26, 27], others reported improvement in adherence to ART with an increase in age [28], and still, others reporting that age was not associated with adherence to ART [29].

In our study, compared to children aged 6–9 years, there were lower odds of non-adherence among children aged 10–14 years. This was supported by a systematic review reported by Hudelson et al. that indicated younger age adolescents had good adherence to ART [10]. School-age children below the age of 10 years are dependent on their caregivers to take medications but with some level of responsibility compared to their corresponding younger ages [30]. Caregivers provide adherence support to their children as young children may lack important skills to do so, and it positively contributes to adherence [14, 31]. However, any problem with the caregiver’s management of their child’s medication, like alcohol intake as found in our study and others, will compromise adherence to ART [16, 17]. Dachew et al. compared adherence to ART between under-five children and other age groups (5–9 and 10–15 years), and observed increases in adherence difficulty with increasing age [27]. Contrary to our finding, a study in Malawi among children (12–18 years) reported that age did not affect adherence to ART [29]. Though not particularly noted in our study, a narrative review reported that older children had poorer ART adherence than any other age group [5]. Still, others reported a mixed effect of age on adherence that its effect varied by sex where only older male children tend to be more adherent [28].

Children’s adherence to ART is multifactorial where the caregiver’s behavior and health status, child-related factors, medication-related factors, and health-system-related factors come into play [14]. Ammon et al. in their review reported some forty-four barriers and twenty-nine facilitators impacting adherence to ART among ALHIV [14]. Among the factors identified were children’s substance use [14] and the caregiver’s use were associated with non-adherence to ART [16, 17]. Primary caregiver’s substance use could contribute to children’s poor adherence by compromising the caregiver’s capacity to continue providing usual care related to keeping medication time. The influence of substance use on medication adherence has been documented elsewhere [32, 33].

Adherence to ART is closely associated with the number of viral copies in blood where an increase in the number of viral copies was observed with the level of suboptimal adherence [34, 35]. In this study, we found that children with high viral copies and/or had detectable viral loads were significantly associated with non-adherence to ART. A similar finding was reported in a previous study that detectable viral load was associated with non-adherence to ART [35].

Research evidence indicates that experiencing side effects associated with a given medication leads to poor adherence [36]. Consistent with previous studies [15, 37, 38], we found that, compared to the Tenofevir-based regimen, non-adherent children had a higher odds of receiving Zidovudine- and Abacabir-based regimens.

The strength of this study was that we estimated non-adherence and identified caregiver-related, child-related, and clinical-related risk factors that affected non-adherence using an unmatched case-control study where data collectors were blinded to children’s adherence status. We also used physician-recorded adherence evaluation from a patient card that we believe could be less biased than self-reported data; we also included potential variables of both the caregiver and their children. However, we did not include variables on household dynamics including quality of the caregiver-child relationship, violence and maltreatment, and health system-related variables which may have potentially affected the observed results. Furthermore, due to the small number of participants per facility, we were not able to report adherence per facility involved or the type of facility. In this case, the adherence reported may reflect the situation of a facility that contributed the largest sample size.

## Conclusion

In this study, we identified that disclosure of HIV status and child being of a younger age reduces the risk of non-adherence to ART, whereas, caregiver’s substance use, ART regimen, and low or no improvements in viral load levels were shown to have increased non-adherence to ART. We, therefore, suggest that providing caregivers with sustained disclosure and adherence support with a particular focus on counseling on the reduction (or cessation) of substance use and setting goals to improve viral load could improve adherence to ART, and hence viral load suppression. Furthermore, we recommend an appropriate selection of first-line ART regimens that could be preferable in terms of possible side effects.

## Data Availability

All data analyzed and pertaining to the findings are presented in this paper.

## List of abbreviations

ART: Antiretroviral Therapy
AOR: Adjusted Odds Ratio
CI: Confidence Interval
COR: Crude Odds Ratio
HIV/AIDS: Human Immunodeficiency Virus/Acquired Immunodeficiency Syndrome
IHRERC: Institutional Health Research Ethics Review Committee
LMICs: Low-and Middle-Income Countries
ND: Not Detectable
OR: Odds Ratio
PLHIV: People Living with HIV
SSA: Sub-Saharan Africa
UNAIDS: The Joint United Nations Programme on HIV and AIDS
WHO: World Health Organization
